# Supplementary material for: Temperate functional niche availability not resident-invader competition shapes tropicalisation in reef fishes
Source: Nat Commun. 2023 Apr 17;14:2181. doi: 10.1038/s41467-023-37550-5 (PMC10110547; doi:10.1038/s41467-023-37550-5)
Supplement: Supplementary file 1 — Supplementary Information [file 41467_2023_37550_MOESM1_ESM.pdf]

Supplementary Information

Title: Temperate functional niche availability not resident-invader competition shapes tropicalisation in reef fishes

Authors: Mark G.R. Miller, James D. Reimer, Brigitte Sommer, Katie M. Cook, John M. Pandolfi, Masami Obuchi, Maria Beger

Supplementary Methods

Drivers of community turnover

To determine the drivers of community species biomass turnover across each latitudinal gradient, we input site by species Bray-Curtis distance matrices into PERMANOVA to test the importance of latitude, longitude, sea surface temperature, chlorophyll-*a* concentration, and site position. Variables were added sequentially (in the order above) to test the contribution of environmental variables with geographical location already accounted for, particularly given the correlation between latitude and sea surface temperature. Monthly mean sea surface temperature (SST) and chlorophyll-*a* concentration (CHL) data were downloaded from the NOAA's Coastwatch website at one and four km resolution respectively. SST data were extracted within 1 km of sites, whereas CHL data were extracted within a 20 km buffer to account for frequent NA pixels. A long-term average of both variables for each site was calculated by taking the median value (to reduce the influence of outlier pixel values) from all months from 2003-2018. Site positions were categorized as either 'offshore island', 'offshore shelf', 'inshore shelf' or 'inshore headland', based on distance to coast and coastal geography.

Interpreting functional groups

To interpret and understand resultant clusters, we fitted classification tree models to our optimal clustering solution, using the traits as explanatory variables [1]. We used the R package *partykit* (version 0.1.16) to fit models, return variable explanatory contribution and construct a node diagram. We also visualised the clusters using wordclouds in R package *ggwordcloud* (version 0.5.0). For wordclouds and classification tree models, missing values were filled via imputation using the R package *mice* (version 3.13.0), and continuous variables cut into discrete categories: body size into six classes following [2]; and depth range into three classes (0-30m, 31-100m, 100m+) (Figs. 2, S2).

Functional Group thermal independence

Prior to tropicalisation analyses we checked whether functional groups were independent of thermal preference. Tropical and temperate thermal guilds represent a coarse binning of species' thermal midpoints, where species with a range of thermal midpoints are contained within each thermal guild. Despite functional groups being clustered based on species traits linked to ecosystem functions, there was the possibility that traits encoded information related to thermal tolerance (e.g., body size). If some functional groups contained 'tropical' species clustered with lower thermal midpoints than others then they would be expected to occur at higher latitude, confounding our comparison of functional group tropicalisation. To test that our functional groups remained independent of thermal bias, we used the thermal midpoint and range dataset of [3]. Instead of thermal midpoints (central point between fifth and 95th percentiles of a species' thermal distribution), we used the 95th percentiles of species' thermal distributions as a measure of contemporary realized upper thermal limits, reflecting species persistence under physiologically, competitive and predatory pressures [3]. This dataset contained thermal midpoints for the majority of tropical thermal guild species in Australia (87%, range between functional groups: 80%-100%) and Japan (84%, range between functional groups: 72%-95%). We tested for differences between functional group thermal midpoints using ANOVA.

Biomass-weighted similarity between tropical and temperate functional niches

Our tropical and temperate functional niches were defined using the 99% kernel utilisation distribution of species occurrences within each site or zone. Our metric of functional overlap therefore did not account for the biomass of the constituent species, which could provide additional explanatory information on niche availability and inter thermal guild competition in the case where some species were abundant and others rare. Specifically, we were interested in whether our metric for competition (functional niche overlap between thermal guilds based on species occurrences) was paired with distance between the biomass centre-points of thermal guilds. For each thermal guild functional niche in PCoA trait space we therefore calculated the corresponding centroid based on species occurrence points weighted by their biomass in each site or zone. We then calculated Euclidean distance (in trait space) between these thermal guild centroids within each site or zone. To test whether increasing functional overlap was paired with distance between the biomass centre-points of thermal guilds we used non-parametric Kendall rank correlation performed at the site level across each latitudinal gradient.

Supplementary Results

Drivers of community turnover

Hierarchical clustering of fish biomass data grouped sampling sites into six zones in both regions (Supplementary Figure S1). PERMANOVA confirmed that site clustering into zones was strongly influenced by latitude in both regions (Japan, pseudo-$F_{(1,22)} = 17.73$, $p = < 0.001$; Australia, pseudo-$F_{(1,18)} = 13.45$, $p = < 0.001$). In Japan, longitude (pseudo-$F_{(1,22)} = 3.96$, $p = 0.002$), sea surface temperature (pseudo-$F_{(1,22)} = 2.46$, $p = 0.029$) and site position (pseudo-$F_{(2,22)} = 2.74$, $p = 0.004$) explained additional variance. In Australia, sea surface temperature (pseudo-$F_{(1,18)} = 2.24$, $p = 0.026$), chlorophyll-*a* concentration (pseudo-$F_{(1,18)} = 9.06$, $p = < 0.001$), and site position (pseudo-$F_{(2,18)} = 2.74$, $p = 0.001$) explained additional variance.

Fish traits and FG identification

An optimal 19 functional groups were identified from the traits (Table S1) of their species within the Japan-Australia transitional fish community (Supplementary Fig. S2). The dendrogram (Supplementary Fig. S2a) faithfully represented the original Gower distance matrix with a mean of absolute deviations of 0.096 between the trait-based distance and cophenetic distance (Supplementary Fig. S3). Only one species, *Cantheschenia grandisquamis* was identified as being potentially placed in the incorrect functional group by the dendrogram (displayed Gower distances of <0.2 with some other species but was grouped in different functional groups from them), which was caused by *Cantheschenia grandisquamis* being the only species with an NA in the diet trait. Nineteen clusters simultaneously maximised the Rand matching index, Jaccard similarity index, and average silhouette width, without creating too many clusters (Supplementary Fig. S4).

To compare tropicalisation between the main functional components of the reef fish community, we selected only large functional groups (≥ 14 species represented in both regions) for further analyses. This approach omitted the detritivore diet class and reef-pelagic and pelagic habitat-association classes.

Analysis of tropicalisation

We assigned species to tropical and temperate guilds based on data obtained from FishBase (www.fishbase.org) and expert opinion by MB (which is informed by fish observations on tropical, subtropical and temperate reefs spanning over 25 years of experience). Tropical functional groups were found to be independent of thermal preference with no significant difference between the realised upper thermal limits of tropical functional groups in Australia (LM: $F_{(8,270)} = 0.94$, $p = 0.485$) or Japan (LM: $F_{(8,236)} = 0.62$, $p = 0.757$) (data obtained from Dr R Stuart-Smith, University of

Tasmania). However, Upper-benthic Planktivores FG2 and Benthic Herbivore/Omnivores FG8 had slightly lower thermal midpoints in both regions (Supplementary Figure S5). The uncertainties about these realised upper thermal limits illustrate the need to use expert opinion to assign the thermal guilds.

Community tropicalisation declined over latitude in both regions but each trend was differentially affected by specific zones (Supplementary Figure S6). In Australia, community-level tropical biomass showed an initial 11-fold decline in the Cold-Tropical Reef zone at 25°S (Tukey: $p = 0.005$) but then returned to a similar level to the Tropical Coral Reef zone at around 27°S (Tukey: $p = 0.98$), southward of this point tropical biomass of zones declined (Tukey: three-fold, $p = 0.04$; eight-fold, $p = 0.01$; and 62-fold, $p = <0.001$). In Japan, community-level tropical biomass did not decline below Tropical Coral Reef zone levels up to a latitudinal threshold of 30.5°N (Tukey: $p = 0.98$, $p = 0.99$), but then showed declines northward of this point (Tukey: four-fold, $p = 0.02$; and 17-fold, $p = <0.001$), except for the Subtropical Reef zone (Tukey: $p = 0.99$).

Biomass-weighted similarity between tropical and temperate functional niches

We found that the majority of functional groups (six) showed no significant relationship between functional niche overlap between thermal guilds based on species occurrences and distance between the biomass centre-points of thermal guilds (Table S2). Five functional groups did not have enough datapoints to test correlation as they contained too many sites where one functional guild was missing (forcing functional overlap to zero and not allowing centroid distance to be calculated). The remaining four functional groups with significant correlations all showed that as functional overlap between thermal guilds increased, the distance between their centres of biomass decreased (Supplementary Figure S7). This finding suggests that for some functional groups, our measure of competition between thermal guilds, using overlap of functional niches based on species occurrences, also captures competition between the most important (biomass-heavy) areas of those functional niches. However, in the context of the greater number of non-significant functional group relationships, no clear conclusion can be drawn and further study of biomass distribution within functional niches in relation to competition is recommended.

Table S1. Trait definitions for subtropical and tropical fishes

Trait (type)	Trait level	What is it?
Body Size (continuous)	4.4-450	Maximum tail length recorded in the core range, in cm
Depth Range (continuous)	3-200	The difference between the deepest and shallowest depths at which a species occurs, in m
Diet (categorical)	Piscivore	Feeds on other fishes
	Predator	Predates on a range of animals, including macrofauna and microfauna predators
	Planktivore	Feeds on phyto- and/ or zooplankton
	Omnivore	Feeds on a mix of flora and fauna
	Corallivore	Obligate corallivore, feeding on scleractinian or octo corals
	Herbivore	Feeds exclusively on plants, includes grazers, farmers, scrapers, and browsers
	Detritivore	Feeds on detritus and plants
Aggregation (ordinal)	Schools	Tends to form schools
	Groups	Usually seen in small groups of 3 to 10 individuals
	Pairs	Typically occur in pairs
	Solitary	Solitary
Position (categorical)	Pelagic	Pelagic species who occasionally venture near a reef
	Reef Pelagic	Pelagic fast swimmers that move well above the reef, but typically found near to a reef
	Upper Benthic	Mobile and changeable distance away from the benthos but with substantial associating with the reef matrix
	Benthic	Living closely associated with the benthic substrate, or swimming within a few cm of the bottom
	Demersal	Living on benthic substrate, usually lying on it
	Sub-benthic	Living within crevices, caves, or holes within the reef matrix

Table S2. Kendall Rank correlation (τ) between functional niche overlap between thermal guilds based on species occurrences and distance between the biomass centre-points of thermal guilds measure at site level across latitudinal gradients. Functional group 16 is omitted due to no temperate functional group.

FG	AUSTRALIA	JAPAN
**15**	ns	τ = -0.30*
**10**	ns	ns
**8**	ns	ns
**2**	ns	τ = -0.58**
**6**		
**12**		
**4**		τ = -0.72***
**1**	τ = -0.54*	

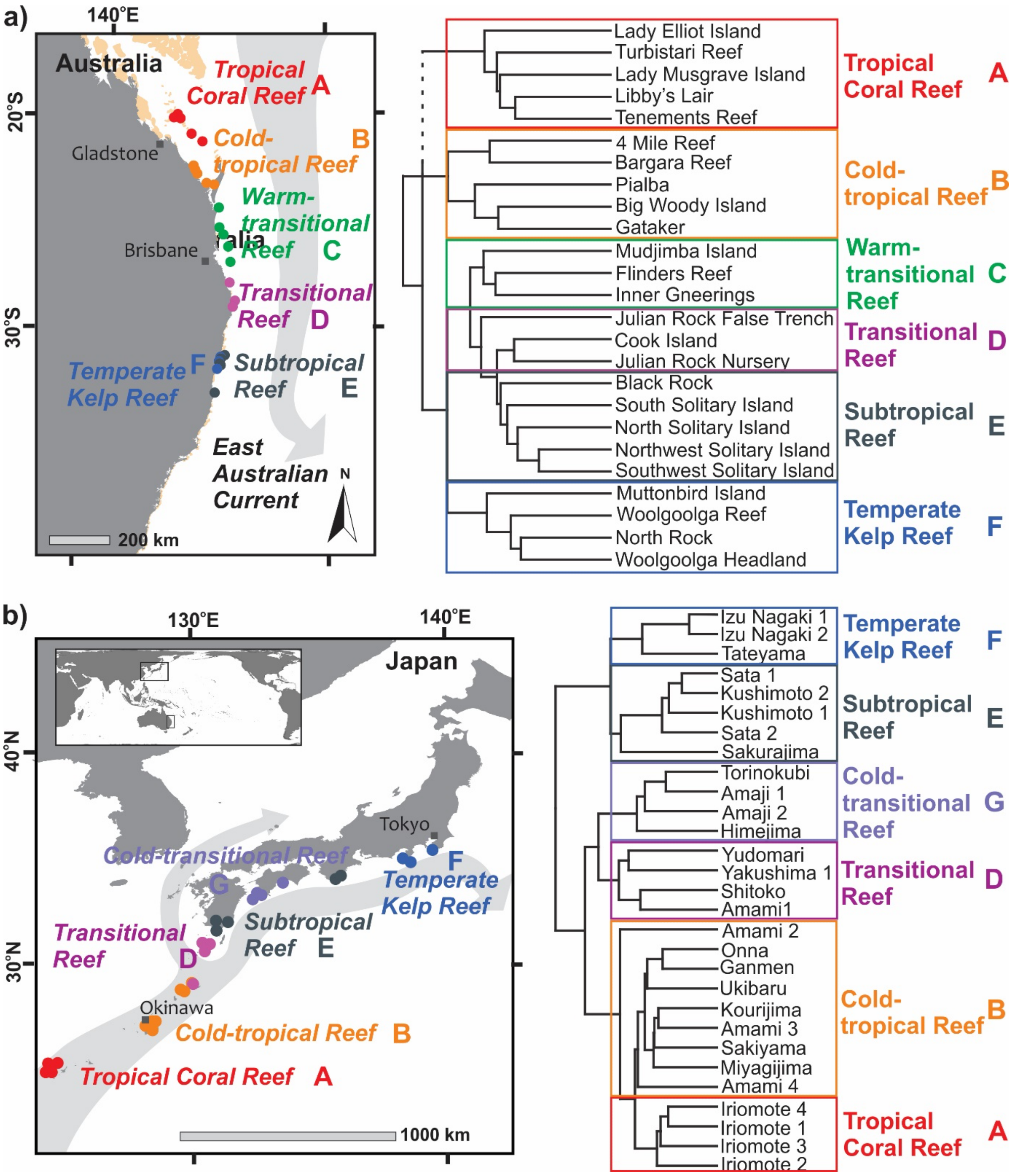

Fig. S1. Map of fish survey sites (dots) grouped into transitional community zones (denoted by colours and capital letters) shown in hierarchical clustering dendrograms derived using mean fish biomass at each site in a) Australia and b) Japan.

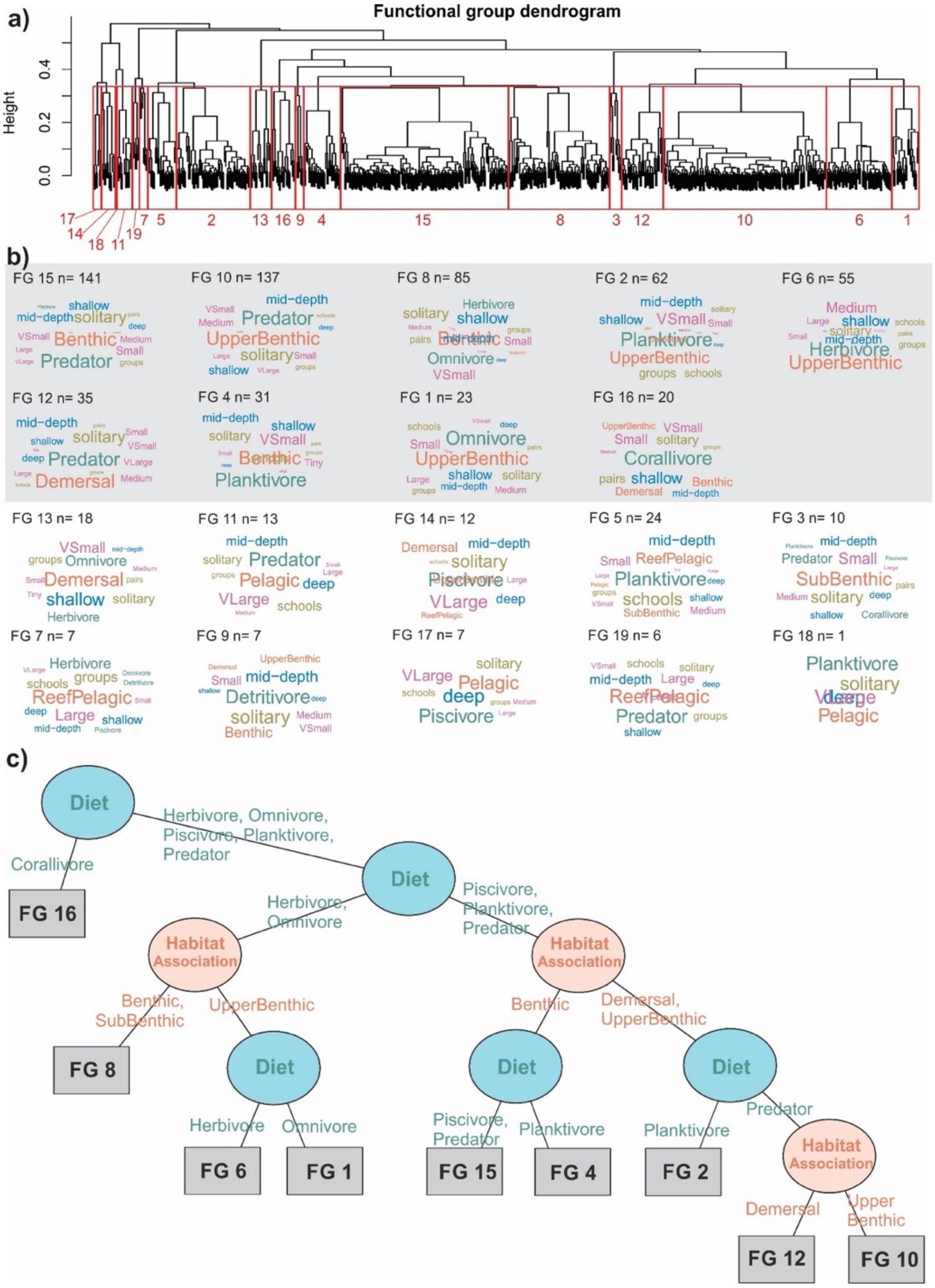

Fig. S2 Functional groups (FG) identified in the Japan-Australia transitional fish meta-community, all groups occur in both regions with the exception of FG18 (Australia). A) Average hierarchical clustering of fish traits produced a dendrogram cut to an optimal 19 functional groups (functional groups numbered below each cluster). B) Functional groups ordered by size (n species) and visualized using wordclouds, where the size of each word indicates its' relative importance in defining the group (size * n occurrence of word in species functional entities). Traits are coloured: diet = green; habitat association = red; body size = purple; aggregation = mustard; depth range = blue. The grey shade shows the nine functional groups carried forward in this study. There are more species per functional group compared to Figure 2 in the main manuscript, as Fig. S2 represents the Japan-Australia transitional fish meta-community in both summer and winter and includes an additional 64 species observed in the Austral summer in Australia that were not observed in the Austral winter biomass data used for modelling tropicalisation trends. C) Conditional inference tree node diagram predicting how traits classify the nine major functional groups, terminal nodes give the functional group number and n species.

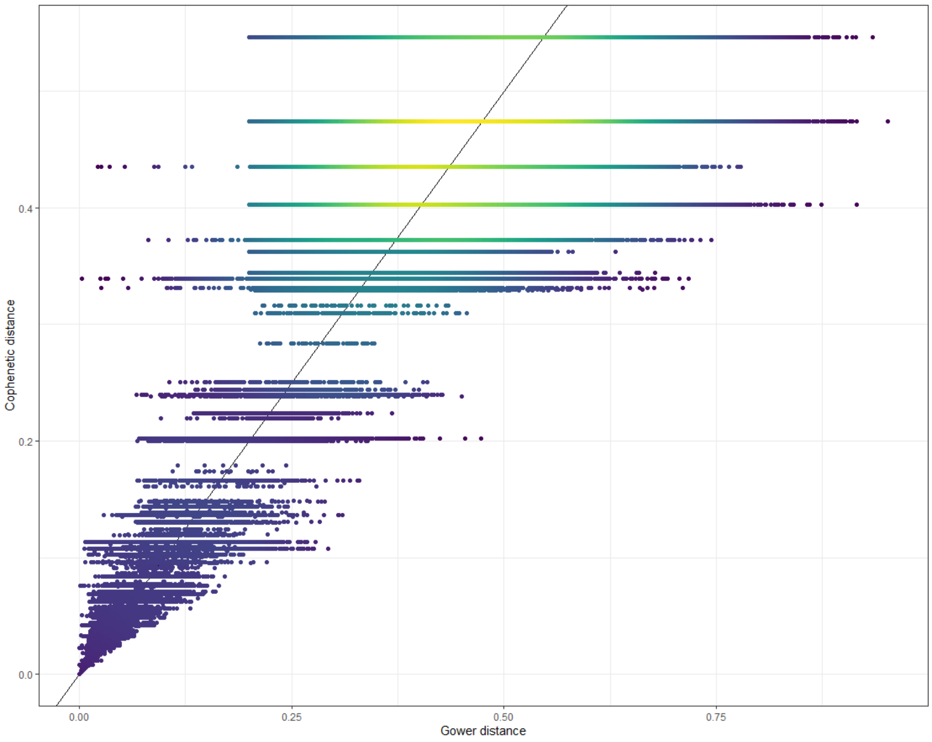

Fig. S3. Shepard diagram of cophenetic distances, as represented in the dendrogram, against the original Gower distances. The point density gradient (blue, low to yellow, high), shows that at higher distances the majority of cophenetic distances still track the 1:1 diagonal line, faithfully representing their Gower counterparts.

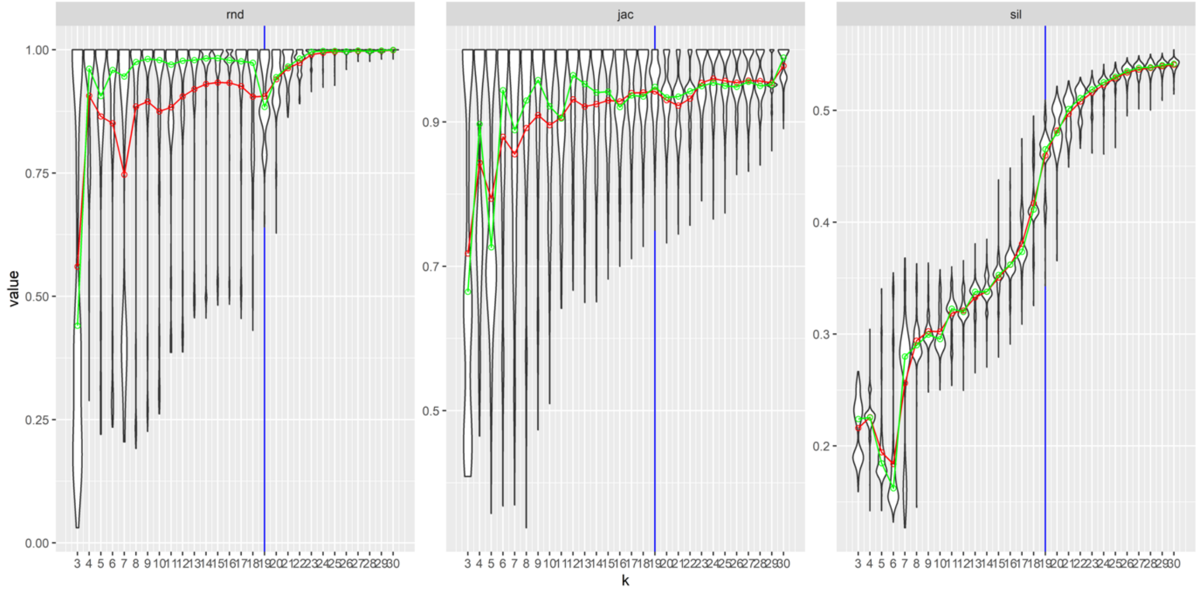

Fig. S4. Choice of optimal number of clusters (functional groups) based on 1000-iteration 5% subsample bootstrap of trait distance matrix. Red points and line are mean values, green points and line are median, rnd = Rand matching Index, jac = Jaccard similarity index, sil = average silhouette width, blue line is optimal number of clusters chosen (19), from 3-30 clusters (k).

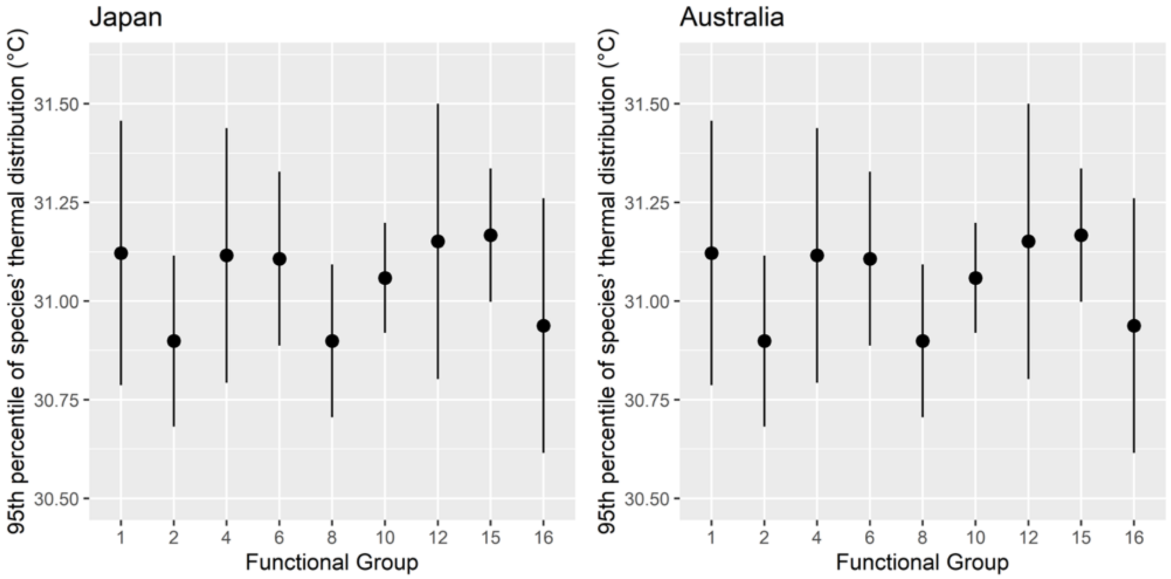

Fig. S5. Realised upper thermal limits (95th percentile of species' thermal distribution) estimated (mean and 95% confidence intervals) for tropical species in functional groups. Functional group tropical thermal midpoints did not differ significantly in Australia (ANOVA: $F_{(8,270)} = 0.94$, $p = 0.485$, n = 279 species) or Japan (ANOVA: $F_{(8,236)} = 0.62$, $p = 0.757$, n = 245 species). Data includes only species where realised upper thermal limits were available (R Stuart-Smith, University of Tasmania).

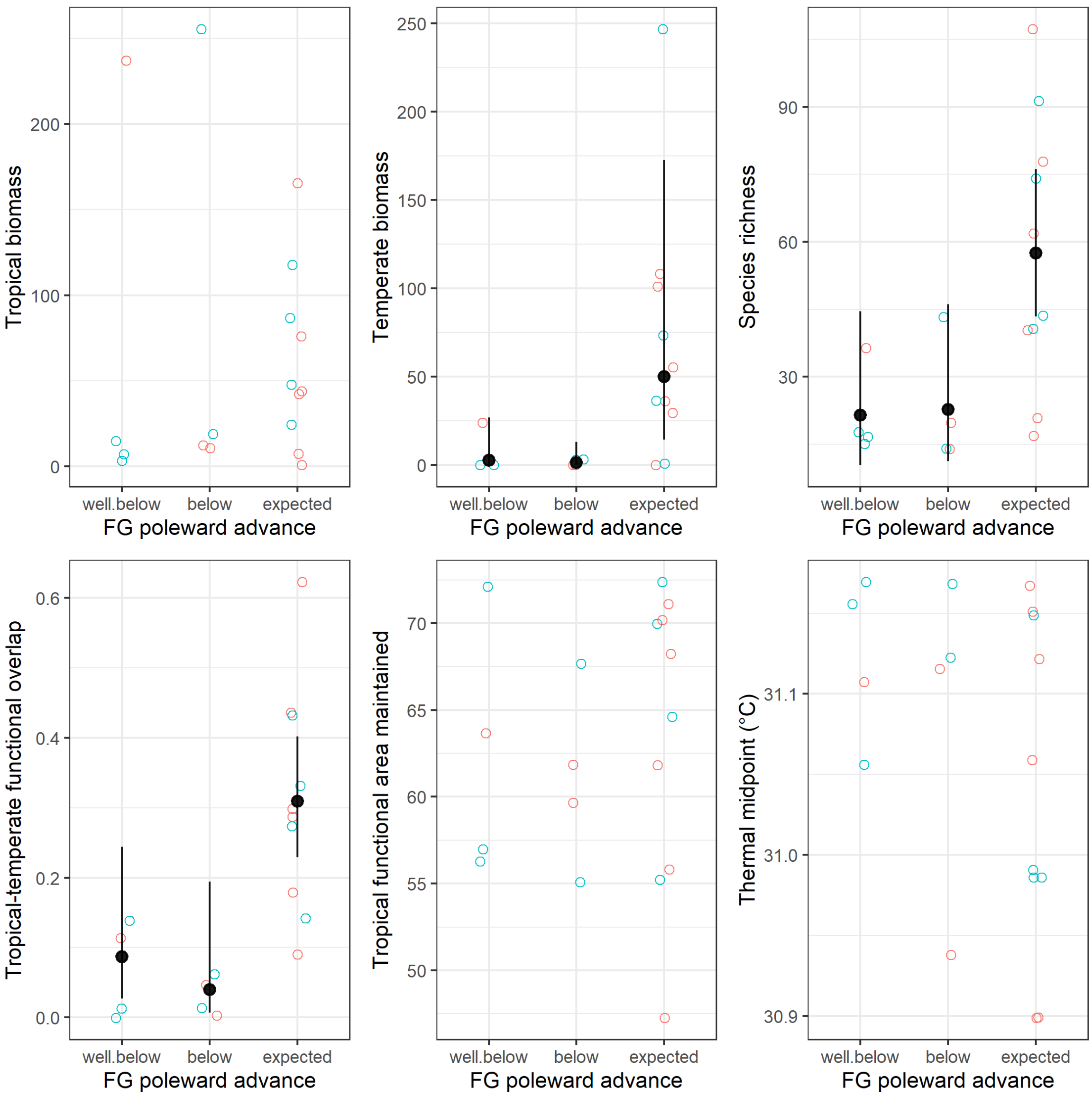

Fig. S6. Drivers explaining differences in the functional group leading-edges (latitude of 20-fold tropical biomass decline). Functional groups were classed into three poleward advance groups based on comparison with the community leading-edge: tropicalising as expected (expected); tropicalising below community expectation (below); and tropicalising well below community expectation (well.below). Hollow points in each plot represent each functional group (pink = Australia, n = 9; blue = Japan, n = 9) and black Mean + 95% confidence intervals are plotted if driver showed significant differences between poleward advance classes. The thermal midpoints in bottom right figure represent realised upper thermal limits (95th percentile of functional groups' thermal distribution). Source data are provided as a Source Data file.

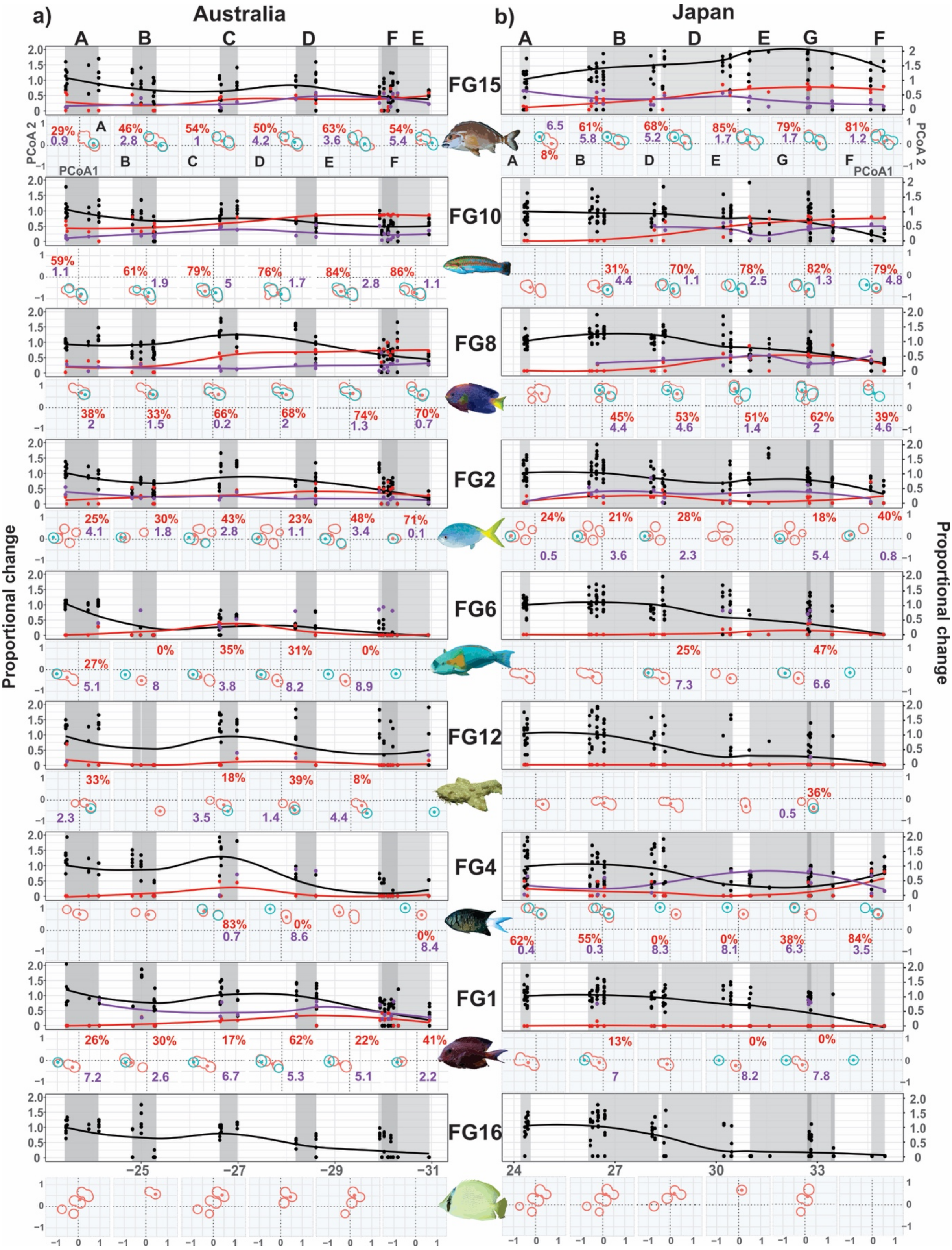

Fig. S7. Changes in functional niche overlap, based on species occurrence and biomass, within nine functional groups (row numbers) over latitude in a) Australia, and b) Japan. Scatter plots show latitudinal change in tropicalisation footprint (proportion of tropical biomass relative to that of the Tropical Coral Reef zone; black), functional niche percent overlap, based on species occurrence, between tropical and temperate thermal guilds (red) and distance between the biomass centre-points of thermal guilds (purple) at site level, with trends estimated by Loess smoothers. Functional trait space plots underlying scatter plots show PCoA ordination of species traits at each of the six zones (A-G). Functional niches of tropical (red) and temperate (light blue) thermal guilds are represented as

polygons (kernel surrounding species' points in trait space) to show functional niche overlap between tropical and temperate thermal guilds (red percentage), and the corresponding coloured point within each functional niche shows the biomass centre-point (with the Euclidean distance between pairs of thermal guild centre-points shown in purple) for each zone. Transitional community zones are coded: A=Tropical Coral Reef, B=Cold-tropical Reef, C=Warm-transitional Reef, D=Transitional Reef, E=Subtropical Reef, F=Temperate Kelp Reef, G=Cold-transitional Reef. Fish icons depict species characteristic for the functional groups, created in CorelDraw 16 from original photographs by the authors. Source data are provided as a Source Data file.

Supplementary References

1. Darling, E. S., Alvarez-Filip, L., Oliver, T. A., Mcclanahan, T. R. & Côté, I. M. Evaluating life-history strategies of reef corals from species traits. *Ecology Letters* **15**, 1378–1386 (2012).
2. Mouillot, D. *et al.* Functional over-redundancy and high functional vulnerability in global fish faunas on tropical reefs. *Proceedings of the National Academy of Sciences* **111**, 13757–13762 (2014).
3. Stuart-Smith, R. D., Edgar, G. J., Barrett, N. S., Kininmonth, S. J. & Bates, A. E. Thermal biases and vulnerability to warming in the world € s marine fauna. *Nature* **528**, 88–92 (2015).
