# Supplementary material for: Temperate functional niche availability not resident-invader competition shapes tropicalisation in reef fishes
Source: Nat Commun. 2023 Apr 17;14:2181. doi: 10.1038/s41467-023-37550-5 (PMC10110547; doi:10.1038/s41467-023-37550-5)
Supplement: Supplementary file 4 — Reporting Summary [file 41467_2023_37550_MOESM4_ESM.pdf]

nature portfolio

Corresponding author(s): Maria Beger, Mark Miller

Last updated by author(s): 2022/07/21

Reporting Summary

Nature Portfolio wishes to improve the reproducibility of the work that we publish. This form provides structure for consistency and transparency in reporting. For further information on Nature Portfolio policies, see our Editorial Policies and the Editorial Policy Checklist.

Statistics

For all statistical analyses, confirm that the following items are present in the figure legend, table legend, main text, or Methods section.

n/a	Confirmed	
☐	☒	The exact sample size (*n*) for each experimental group/condition, given as a discrete number and unit of measurement
☐	☒	A statement on whether measurements were taken from distinct samples or whether the same sample was measured repeatedly
☐	☒	The statistical test(s) used AND whether they are one- or two-sided<br>*Only common tests should be described solely by name; describe more complex techniques in the Methods section.*
☐	☒	A description of all covariates tested
☐	☒	A description of any assumptions or corrections, such as tests of normality and adjustment for multiple comparisons
☐	☒	A full description of the statistical parameters including central tendency (e.g. means) or other basic estimates (e.g. regression coefficient) AND variation (e.g. standard deviation) or associated estimates of uncertainty (e.g. confidence intervals)
☐	☒	For null hypothesis testing, the test statistic (e.g. *F*, *t*, *r*) with confidence intervals, effect sizes, degrees of freedom and *P* value noted<br>*Give P values as exact values whenever suitable.*
☒	☐	For Bayesian analysis, information on the choice of priors and Markov chain Monte Carlo settings
☒	☐	For hierarchical and complex designs, identification of the appropriate level for tests and full reporting of outcomes
☐	☒	Estimates of effect sizes (e.g. Cohen's *d*, Pearson's *r*), indicating how they were calculated

Our web collection on statistics for biologists contains articles on many of the points above.

Software and code

Policy information about availability of computer code

Data collection	No software was used for data collection
Data analysis	CorelDraw 16 &20, R 4.1.0, vegan (version 5.2.7), cluster (version 2.1.2), clue (version 2.1.0), nlme (version 3.1-157), Ade4 (version 1.7-17), adehabitatHR (version 0.4.19), partykit (version 0.1.16), ggwordcloud (version 0.5.0), mice (version 3.13.0), ggplot2 (version 3.3.3)

For manuscripts utilizing custom algorithms or software that are central to the research but not yet described in published literature, software must be made available to editors and reviewers. We strongly encourage code deposition in a community repository (e.g. GitHub). See the Nature Portfolio guidelines for submitting code & software for further information.

Data

Policy information about availability of data

All manuscripts must include a data availability statement. This statement should provide the following information, where applicable:

- Accession codes, unique identifiers, or web links for publicly available datasets
- A description of any restrictions on data availability
- For clinical datasets or third party data, please ensure that the statement adheres to our policy

Data for species used and their traits are included in the Supplementary Material. The species abundance and source data are available upon request. Code and data are available from GitHub at: https://github.com/lark-gorilla/coral_fish/blob/master/code.R.

Human research participants

Policy information about studies involving human research participants and Sex and Gender in Research.

Reporting on sex and gender	N/A
Population characteristics	N/A
Recruitment	N/A
Ethics oversight	N/A

Note that full information on the approval of the study protocol must also be provided in the manuscript.

Field-specific reporting

Please select the one below that is the best fit for your research. If you are not sure, read the appropriate sections before making your selection.

☐ Life sciences ☐ Behavioural & social sciences ☒ Ecological, evolutionary & environmental sciences

For a reference copy of the document with all sections, see nature.com/documents/nr-reporting-summary-flat.pdf

Ecological, evolutionary & environmental sciences study design

All studies must disclose on these points even when the disclosure is negative.

Study description	The study is focused upon changes in functional niches of reef fishes over a latitudinal gradient and follows two themes: 1) how these functional niches change and overlap in multidimensional trait space; and 2) how the biomass of fishes assigned to functional niches change. The latitudinal gradient provides the backbone for the study to investigate tropicalization and fish are either assigned to tropical or temperate thermal guilds.<br>Nineteen functional groups of species were identified of which nine had sufficient biomass data to be analysed for topicalization trends. Change in the biomass of tropical species within these nine functional groups as compared over 25 sites in Australia and 29 sites in Japan, representing the latitudinal gradient. To detect statistical differences in tropicalization rates between functional groups, the sites were grouped into six transitional zones. The nine functional groups (split into their two thermal guilds) had their functional niches represented in two-dimensional trait space at individual sites and at transitional. At both levels, the contraction of tropical functional niches was measured and the overlap between tropical and temperate niches was measured. Both of these metrics were correlated with tropicalization trends.
Research sample	All non-cryptic reef fishes at 54 (29 in Japan and 25 in Australia) sites along a tropical to temperate environmental gradient, targetting coral communities. Non-cryptic fishes were chosen for ease of identification and because of time-limitations on a dive that means very detailed assessments of cryptic fauna is not feasible. All individuals of any size and sex were recorded. Each sample represents the fish community per site.
Sampling strategy	Standard underwater visual census of fishes on belt transects, where one observer (MB) counted and estimated each fish seen on 3 to 5 replicate transects per site (3 in Japan, 5 in Australia), with an estimated width of 5m and a measured length of 25 m (Japan, some sites in Australia) and 50m (Australia), length was determined by habitat availability. 8 m depth, except where coral communities were shallower (Tateyama 3m, Woolgoolga Headland, Muttonbird Island 5 m)
Data collection	Fish visual census conducted by Maria Beger, with length estimates to the nearest cm. Transect lengths were measured with 50m measuring tapes.
Timing and spatial scale	Japan: June 2015, 2016<br>Australia: 2010, 2011, 2012, 2013, 2016, 2017, 2018, 2019<br>Each sampling period lasted approximately 4 weeks per year and country.<br>Spatial scales (see exact locations below):<br>Japan: Iriomote to Tatayama (near Tokyo)<br>Australia: Southern Great Barrier Reef to Southwest Rocks (in New South Wales)
Data exclusions	No exclusions
Reproducibility	Core data are submitted alongside the paper as appendices, remaining data are available upon request. The code necessary to run all analyses in the paper (and reliant upon the submitted or requested data) is publicly available via Github and can be downloaded and run without restriction in free open source software R.
Randomization	Our study relied on deliberate grouping of fish species into functional groups based on five traits (diet, habitat association, body size, aggregation, and depth range) linked to species effects on ecosystem functioning, and sites grouped into transitional zones based their fish communities. Both of these groupings were achieved via hierarchical clustering. An important grouping consideration was

to check whether the created functional groups were independent of thermal preference. Despite functional groups being clustered based on species traits linked to ecosystem functions, there was the possibility that traits encoded information related to thermal tolerance (e.g., body size). If some functional groups contained 'tropical' species clustered with lower thermal midpoints than others then they would be expected to occur at higher latitude, confounding our comparison of functional group topicalization. An external thermal dataset was sourced to confirm independence.

Blinding

Blinding was not necessary in our study

Did the study involve field work? ☒ Yes ☐ No

Field work, collection and transport

Field conditions

Underwater visual census on coral communities, counting and sizing fishes, sheltered reefs, ca 8m deep. Temperature range from 18 to 30 degrees water temperature.

Location

Region Site name Lat Long
AUS1 Southern Great Barrier Reef Heron Island - Wistari -23.45883 151.86971
AUS2 Southern Great Barrier Reef Heron Island - Libby's Lair -23.43458 151.93364
AUS3 Southern Great Barrier Reef CORALGARDENS -23.44729 151.91197
AUS4 Southern Great Barrier Reef Heron Island - Tenements 1 -23.43274 151.92926
AUS5 Southern Great Barrier Reef Heron Island - Tenements 2 -23.43258 151.93089
AUS6 Southern Great Barrier Reef Lady Musgrave -23.90603 152.38695
AUS7 Southern Great Barrier Reef Lady Elliott Island -24.115003 152.709506
AUS8 Near Gladstone Ethel Rocks -23.97036667 151.6296333
AUS9 Near Gladstone Pancake Creek -24.02203333 151.7397333
AUS10 Bundaberg Stringers -24.47775 152.0338333
AUS11 Bundaberg Bargara -24.80951 152.47176
AUS12 Bundaberg Barolin Rocks -24.8826 152.4901167
AUS13 Bundaberg 4 Mile Reef -24.99149 152.55405
AUS14 Hervey Bay Gatakers -25.2476 152.79913
AUS15 Hervey Bay Gables -25.248067 152.8301
AUS16 Hervey Bay Big Woody -25.26352 152.93697
AUS17 Hervey Bay Pialba -25.27288 152.84497
AUS18 Hervey Bay Scarness Pier -25.28041667 152.8564667
AUS19 Hervey Bay Torquay -25.28058333 152.8641167
AUS20 Hervey Bay Round Island -25.28521667 152.9242333
AUS21 Hervey Bay Little Woody (N end) -25.3149 153.0164833
AUS22 SE of Fraser Island Wolf Rock -25.9166667 153.2
AUS23 Sunshine Coast Mudjimba Island -26.61614 153.11296
AUS24 Sunshine Coast Inner Gneering Shoals -26.64858 153.18344
AUS25 Outer Moreton Bay Flinders Reef -26.97765 153.48412
AUS26 Outer Moreton Bay Hendersons Shoals -27.13161 153.47815
AUS27 Outer Moreton Bay Flat Rock -27.39306 153.5522
AUS28 Inner Moreton Bay Peel Island -27.4952 153.3327
AUS29 Inner Moreton Bay Goat Island -27.5156 153.383
AUS30 Inner Moreton Bay Myora Reef -27.47228 153.405833
AUS31 Tweed Cook Island -28.19627 153.57632
AUS32 Byron Bay Julian Rocks Nursery -28.61087 153.62809
AUS33 Byron Bay Julian Rocks False Trench -28.61257 153.62863
AUS34 Solitary Islands Marine Park - Coffs Harbour North Solitary Island -29.927723 153.389619
AUS35 Solitary Islands Marine Park - Coffs Harbour North Rock -29.97339 153.25717
AUS36 Solitary Islands Marine Park - Coffs Harbour Northwest Solitary Island -30.018969 153.269667
AUS37 Solitary Islands Marine Park - Coffs Harbour Woolgoolga Headland -30.10816 153.21054
AUS38 Solitary Islands Marine Park - Coffs Harbour Woolgoolga reef -30.09374 153.20561
AUS39 Solitary Islands Marine Park - Coffs Harbour Southwest Solitary Island -30.159215 153.22809
AUS40 Solitary Islands Marine Park - Coffs Harbour South Solitary Island -30.20478 153.26515
AUS41 Solitary Islands Marine Park - Coffs Harbour Muttonbird Island -30.3033 153.15112
AUS42 South West Rocks Black Rock -30.948371 153.076078

Access & import/export

All samples were collected with minimal disturbance, we conducted non-invasive observational sampling.
Permits were obtained.
NSW DPI SIMP 2010/001; P10/0024-2.0; 2016/002v2
Queensland Gov DPI 140700
Dept of Env & Resource Management, Queensland, Great Sandy Marine Park: QS2010/GS036
GBRMPA exemption permit University of Queensland UQ002/2017, GBRMPA G18/40615.1
Animal ethics were required via the University of Queensland, project ID 193/10

	In Japan, no permits are required for observational studies, all site specific entities (e.g. fisheries management groups) were consulted where appropriate.
Disturbance	Observational data collection over short periods of time caused minimal disturbance, diving involved no touching of the substrate by divers.

Reporting for specific materials, systems and methods

We require information from authors about some types of materials, experimental systems and methods used in many studies. Here, indicate whether each material, system or method listed is relevant to your study. If you are not sure if a list item applies to your research, read the appropriate section before selecting a response.

Materials & experimental systems

n/a	Involved in the study
☒	☐ Antibodies
☒	☐ Eukaryotic cell lines
☒	☐ Palaeontology and archaeology
☒	☐ Animals and other organisms
☒	☐ Clinical data
☒	☐ Dual use research of concern

Methods

n/a	Involved in the study
☒	☐ ChIP-seq
☒	☐ Flow cytometry
☒	☐ MRI-based neuroimaging
